# Serrated Adenoma of Gastric Antrum: Alteration of Mucin Expression Profile and its Role in Carcinogenesis

**DOI:** 10.4021/gr2009.05.1294

**Published:** 2009-05-20

**Authors:** Yesim Gurbuz, Cem Aygun, Gupse Turan

**Affiliations:** a Department of Pathology, Kocaeli University Medical Faculty, Umuttepe Kampusu Kocaeli-Turkey; b Department of Gastroenterology, Kocaeli University Medical Faculty, Umuttepe Kampusu Kocaeli, Turkey

**Keywords:** Serrated adenoma, Gastric polyp, Immunohistochemistry

## Abstract

Serrated adenomas usually occur in colon, the gastric localization is extremely rare. These polyps have their own carcinogenetic pathway with microsatellite instability. In this report, we present a serrated adenoma localized in gastric antrum with four control endoscopies and biopsies. Immunohistochemical panel of MUC1, MUC2, MUC5AC, and MUC6 was applied to the biopsies. Serrated component, MUC 2 expression increased but goblet cells and MUC5AC expression decreased in follow-up biopsies. This lesion probably was originated from a stem cell that had the potential of differentiation in gastric and intestinal way. This might result an incomplete metaplasia for both colon and stomach. Such lesions which originate from either colon or gastric mucosa may be precancerous and their carcinogenetic pathway may not represent its original organ.

## Introduction

Serrated adenomas are mixed hyperplastic and adenomatoid polyps that are characterized by prominent serrated appearance with high columnar eosinophilic cells [1]. They are found most commonly in colon and they constitute 0.6% to 6% of all intestinal adenomas [2, 3]. Patients with serrated adenomas may range in age from 15 to 88 years with a mean of 63 years. Lesions may be solitary or may accompany familial adenomatosis coli or serrated adenomatosis poliposis syndromes [4]. These lesions became recently popular because of a number of reports that insisted on their precancerous potential with a genetical pathway independent of APC mutations [5-10].

The ordinary place of serrated adenoma is sigmoid colon or rectum but also it is reported in small bowel in a familial adenomatous poliposis syndrome [11]. Gastric localization is quite rare with only six cases reported in English literature [12, 13]. In this report, we will present a serrated adenoma localized in gastric antrum which had follow up biopsies and we will discuss the altered mucin expression of the lesion which may have a possible role in its carcinogenetic pathway. The alterations in tumor morphology and mucin expression profile could make us to understand the nature of this entity better.

## Case report

**Figure 1:**
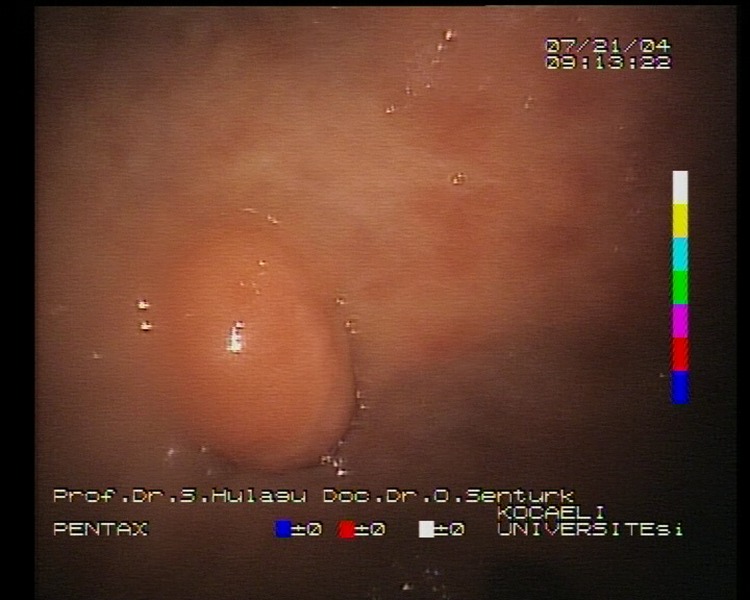
Endoscopic view of sessile polyp localized in prepyloric region.

A 57-year-old woman applied to our gastroenterology outpatient-clinics with intermittent nausea and vomiting in August 1994. A sessile polypoid mass of 15 X 15 mm dimension was observed in prepyloric region during the endoscopic examination (Fig 1). The patient refused polyp resection or biopsy at that time and 6 years later she had applied to the gastroenterology department for control. Later on she had four follow-up endoscopies between 2-year intervals. The polyp in prepyloric region persisted without any progression during this period. In the second biopsy the polyp was exulcerated under endoscopy and this finding was confirmed by histopathology.

Totally, 12 biopsies were evaluated from the target lesion (3 biopsies from first, 4 biopsies from second, 2 biopsies from third, 3 biopsies from fourth endoscopy). In order to evaluate the progress of this polyp, we calculated the percentage of intestinal metaplastic component, the serrated component and also the intensity of mucin expression profile of each biopsy and took an average for each endoscopic biopsy set. Percentage of identifiable intestinal metaplastic component and serrated component for each biopsy material were calculated, so we were able to take an average for each biopsy. Goblet cells were accepted as the hallmarks of intestinal metaplasia. For immunohistochemical evaluation we calculated the percentage of immunoreactive mucosal component and took an average percentage for each biopsy.

An appropriate paraffin block was selected from four follow-up biopsies for immunohistochemical examination and a panel of MUC1 (E29 Neomarkers Fremont CA, USA), MUC2 (M53 Neomarkers Fremont CA, USA), MUC5AC (45M1 Neomarkers Fremont CA, USA), and MUC6 (MCN6.01 Neomarkers Fremont CA, USA) was applied. Histochemical stain of PAS-Alcian Blue Ph:2,5 was also applied to slides.

**Figure 2:**
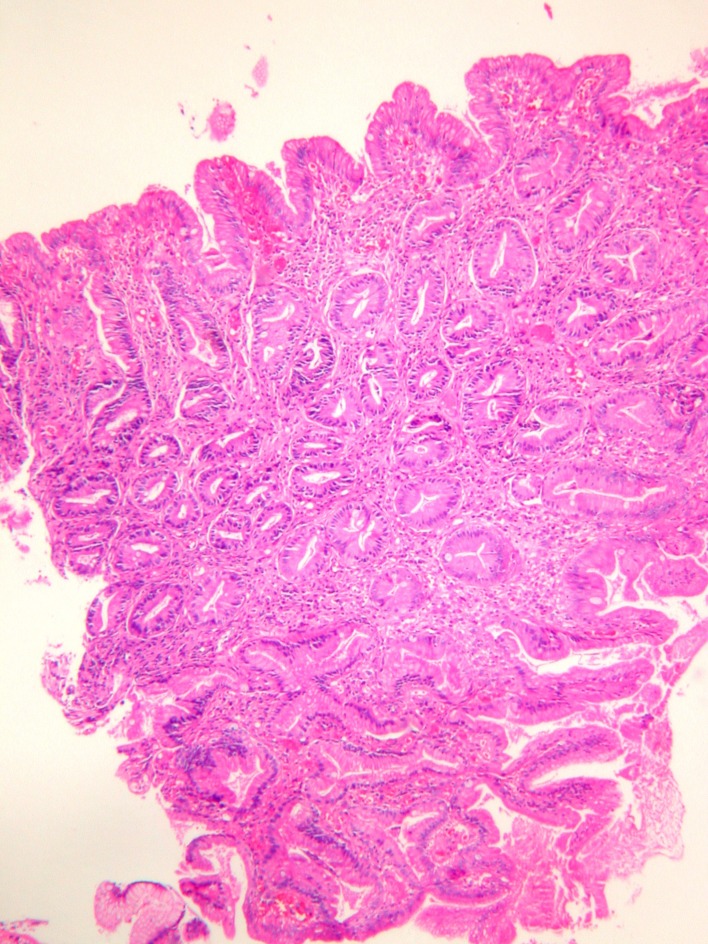
Serrated glands with a few goblet cells composed of high columnar eosinophilic cells in the serrated component of the adenoma (HE X100).

In microscopic examination, the first biopsy was composed of distorted and hyperplastic glandular structures with fibrovascular stroma. Intestinal metaplasia and a few glands composed of eosinophylic high columnar cells with slightly enlarged nucleus were observed and therefore lesion was diagnosed as hyperplastic polyp with intestinal metaplasia. Later in the second and third biopsy sets the glandular distortion and cellularity increased. In the last biopsy the polypoid mass had short villous proliferations composed of high columnar eosinophilic cells on the mucosal surface. The stroma was fibrotic and a few inflammatory cells were observed (Fig. 2). The component composed of crowded glands with large and oval nuclei, eosinophylic cytoplasm with distorted starry shaped glands increased progressively during the follow up biopsies (Fig. 2) (Table 1). Mild cellular atypia was observed in a few cells in the third and fourth biopsy set. We counted goblet cells in ten-high power fields in their most frequently observed localizations. The number of goblet cells also decreased during the follow up. Surrounding gastric mucosa was detected in only 1 biopsy from the third endoscopy and there was chronic antral gastritis without *Helicobacter Pylori* colonization.

**Table 1:** Histopathologic examination and MUC expression percentages of the follow-up biopsies of the serrated adenoma.

Biopsy No.	Serrated component	Intestinal metaplasia	Goblet cells	MUC1	MUC2	MUC5AC	MUC6
1	3%	21.6%	80	100%	37.5%	95%	66%
2	23%	6.6%	60	100%	50%	70%	66%
3	50%	13%	68	100%	70%	70%	100%
4	63%	6.6%	40	90%	75%	10%	100%

The immunoreactivity percentages are calculated for all biopsy specimens not for a special histopathological component

**Figure 3:**
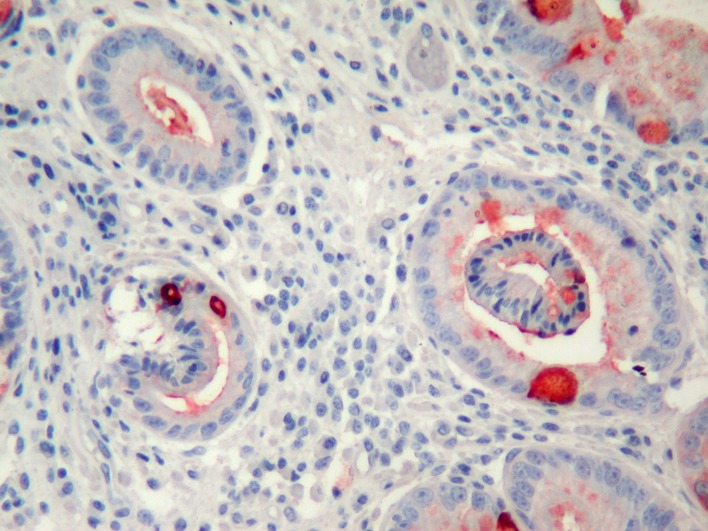
MUC2 expression commonly in goblet cells but also slightly in other cells (immunohistochemistry MUC2 X200).

**Figure 4:**
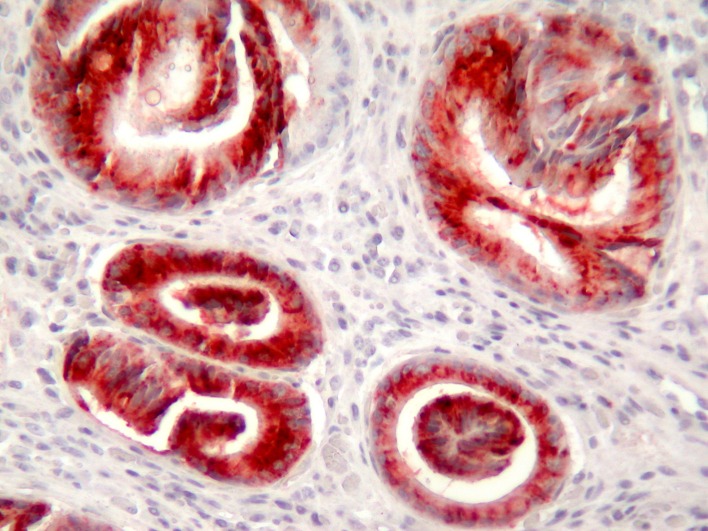
MUC5AC expression commonly in the distorted glands (immunohistochemistry MUC5AC X200).

In PAS-Alcian Blue stain, eosinophilic columnar cells were stained purple and goblet cells were stained deep blue. PAS positive cells were decreased in surface epithelium during the follow-up. MUC1 immunoreactivity was observed in all cells. MUC 2 was observed commonly in goblet cells but also in non-goblet cells (Fig. 3), MUC5AC was observed more strongly in surface epithelium, but weakly in eosinophilic and goblet cells (Fig. 4). MUC6 was observed in all cell types. Co-expression of MUC1, 2, 5AC and 6 was observed frequently.

The number of biopsies, percentage of identifiable intestinal metaplastic component, serrated component and the immunoreactivity with MUC1, MUC2, MUC5AC and MUC6 are summarized in Table 1.

## Discussion

The diagnosis of serrated adenoma is still controversial. Existence of hyperplastic and adenomatoid epithelium in the same polyp is used as diagnostic criteria by some authors. Other groups classify this entity as unmixed polyp and look for serrated type epithelium with glandular distortion and cells with eosinophilic cytoplasm [1, 4]. In this study, we confirmed our diagnosis with Bariol’s histopathological criteria [1]. Serrated pattern was more than 20% in the last 3 biopsies. There was also an involvement of superficial epithelium with cellular and architectural atypia, horizantal crypt alignment, mucin depletion and surface epithelial tufting. In our case villous component was not prominent but villous configuration is not a rule and these lesions may have also tubular, and tubulovillous architectures [14].

An important question about serrated adenomas might be whether the adenomatous component develop from hyperplastic polyps or hyperplastic features are a separate entity in neoplastic polyp [4]. From genetical stand point of view, serrated polyps seem to be heterogeneous, their p53 and bcl-2 expressions are indeed intermediate between those of pure hyperplastic and adenomatous polyps [15]. Inhibition of apoptosis, aberrant crypt formation, hyperplastic polyp, mixed adenoma, serrated adenoma and adenocarcinoma seems to be the pathway [6, 8, 11, 16]. According to our follow-up data we can also clearly state that serrated components might originate from hyperplastic polyps and progress by time.

There are a number of recent reports that insisted on precancerous potential of hyperplastic and serrated polyps with microsatellite instability (MSI) pathway [5, 7, 17-19]. The reported serrated adenoma cases of stomach were also accompanying gastric adenocarcinoma and MSI was also reported in gastric carcinomas [20, 21]. Adenomatous and hyperplastic dysplasias are types of gastric mucosal displasia. Adenomatous dysplasia is similar to its counterpart in colon and originates from complete intestinal metaplasia. The hyperplastic one is composed of dominantly one layer epithelium with large nuclei, prominent nucleoli with eosinophilic cytoplasm similar to serrated epithelium and they originate from incomplete intestinal metaplasia [9, 22]. Serrated type epithelium may be a form of incomplete gastric metaplasia of the intestine. Gastric differentiation of hyperplastic polyps and serrated adenomas were also reported [22]. Therefore, it is not surprising to be precancerous for such an immature tissue and the tumors that arise from that lesion may not show colonic phenotype.

As we observed in our case, mucin expression profiles of hyperplastic polyps and serrated adenomas are similar with upregulation of goblet cell MUC2, reduction of intestinal mucin MUC4 and neoexpression of gastric mucin MUC5AC [2]. Gastric differentiation in mucin expression profile suggests that these tumors may originate from stem cells that can differentiate both gastric and intestinal direction similar to intestinal metaplasia of gastric mucosa [23, 24]. In our case we had the chance to observe the progression of a serrated adenoma and found an increase in serrated component. The reduction in the number of goblet cells and increase in MUC2 immunoreativity was also compatible with incomplete intestinal metaplasia. On the other hand progressive decrease in MUC5AC expression and gastric and intestinal mucin coexpression could be related with increase in the stem cell clone.

Serrated adenoma of the gastric mucosa made us to think about the origin of this lesion again. Although this entity is known to be a lesion of colonic mucosa, the main principals of its histogenetical origin may be the same with its gastric counterpart. The origin of the lesion may be a hyperplastic polyp that is originated with apoptosis inhibition. Later a stem cell clone that has the potential of differentiation in gastric and intestinal way may grow. This may be a form of incomplete intestinal metaplasia in stomach or incomplete gastric metaplasia in colon. Increase in serrated component and MUC2 expression and decrease in MUC5AC expression in our case may show the predominance of the immature clone. As a conclusion, a serrated lesion that originates from either gastric mucosa or colon may be precancerous and their carcinogenetic characteristics may not show their original organ.
